# The influence of the pore shape on the bulk modulus and the Biot coefficient of fluid-saturated porous rocks

**DOI:** 10.1038/s41598-020-75979-6

**Published:** 2020-11-03

**Authors:** A. P. S. Selvadurai, A. P. Suvorov

**Affiliations:** 1 McGill University, Montreal, QC H3A 0C3 Canada; 2 Department of Applied Mathematics, Moscow State University of Civil Engineering (MGSU), Moscow, Russia

**Keywords:** Geodynamics, Geology, Geophysics, Hydrogeology, Environmental sciences, Solid Earth sciences, Engineering, Materials science

## Abstract

Fluid-saturated rocks are multi-phasic materials and the mechanics of partitioning the externally applied stresses between the porous skeleton of the rock and the interstitial fluids has to take into consideration the mechanical behaviour of the phases. In these studies the porosity of the multi-phasic material is important for estimating the multi-phasic properties and most studies treat the porosity as a scalar measure without addressing the influence of pore shape and pore geometry. This paper shows that both the overall bulk modulus of a porous medium and the Biot coefficient depend on the shape of the pores. Pores with shapes resembling either thin oblate spheroids or spheres are considered. The Mori–Tanaka and the self-consistent methods are used to estimate the overall properties and the results are compared with experimental data. The pore density and the aspect ratio of the spheroidal pores influence the porosity of the geomaterials. For partially saturated rocks, the equivalent bulk modulus of the fluid–gas mixture occupying the pore space can also be obtained. The paper also examines the influence of the pore shape in estimating the Biot coefficient that controls the stress partitioning in fluid-saturated poroelastic materials.

## Introduction

The main purpose of this paper is to examine the important role that the pore shape plays in controlling the overall elastic moduli of fluid-saturated porous media. Several researchers have used information on the microstructure of a porous medium, such as the shape of the pore, to estimate the overall properties of the soil. For example, Bary^1^ considers pores with a spherical shape and Giraud et al.^2^ model the pores as thin oblate spheroids with a very small aspect ratio. Gruescu et al.^3^ noticed that the theoretical prediction of the overall thermal conductivity of the porous medium matches the experimental values if the geometric aspect ratio (i.e. the smallest to the largest pore dimension) of the pores is equal to about 1/20. Mavko et al.^4^ considered a porous system consisting of two types of pores: spherical and oblate spheroids. If information on the porosity and shape of the pores is given, we can use one of the effective media approaches to estimate the overall properties. The contributions in this area are too many to provide a detailed record of the advances. The reader is referred to the review articles and texts by Paul^5^, Budiansky^6^, Hashin^7,8^, Budiansky and O’Connell^9^, Christensen^10^, Mura^11^, Kachanov^12^, Nemat-Nasser and Hori^13^, Buryachenko^14^ and Mavko et al.^4^. The most frequently used effective medium approaches are the Mori–Tanaka (Mori and Tanaka^15^; Bary^1^; Giraud et al^2^) and the self-consistent (Mavko et al.^4^) methods. In the Mori–Tanaka approach, each inhomogeneity (an inclusion or a pore) is considered to be embedded within a large volume of the material constituting the matrix phase, whereas in the self-consistent method each pore is embedded within a volume of the material having as yet unknown properties. By exploiting this embedding technique, a complicated problem of interaction between closely spaced inclusions is replaced by a simpler problem in which each inhomogeneity interacts with only a large volume of so-called comparison material in which the inhomogeneity is embedded.

The paper first presents estimates of the overall bulk modulus of a three-phase porous medium consisting of the solid phase (matrix), fluid phase and gas phase. The case of a fully-saturated porous medium can be obtained as a special case of this system by setting the saturation equal to unity. After obtaining an estimate of the overall bulk modulus, the Biot coefficient can be determined analytically. By matching the analytical estimate with the experimentally measured Biot coefficient for several rocks, we can then determine the shape of the pores that correlate with experimental data.

The proposed estimates can also be used to find the equivalent bulk modulus of the fluid–air mixture in the partially saturated porous material. We note that if the fluid–gas mixture in the porous material is treated as a two-phase system, then the equivalent compressibility of the mixture is given by the Hashin–Shtrikman^16^ bound $C_{fa} = C_{a} (1 - S) + C_{f} S$. The Hashin–Shtrikman bounds of the overall bulk modulus of the isotropic two-phase system are obtained when the shear modulus of the constituents is set equal to zero. Since the compressibility of the gas $C_{a}$ is much larger than the compressibility of the fluid $C_{f}$, the compressibility of the air dominates the solution if the saturation $S$ is smaller than unity.

In this paper, the fluid–gas mixture is considered to be part of a three-phase porous medium that includes the solid phase. A different estimate of the equivalent fluid–gas compressibility that depends on the shape of the pores can then be obtained. Other estimates of the compressibility of the fluid–gas mixture are available. For example, Vgenopoulou and Beskos^17^ considered the equivalent compressibility as a function of the depth below the ground surface, and, at large depths, the equivalent compressibility of the mixture becomes equal to the compressibility of the fluid when saturation is close to unity. A recent study by Selvadurai and Ichikawa^18^ also provides estimates for the effective compressibility of a fluid–gas mixture; it was observed that the air voids content in a porous medium has a significant influence on the experimental results governing hydraulic pulse tests used to estimate the permeability characteristics of low permeability rocks. Similar conclusions have also been reported by Selvadurai and Najari^19,20^.

## Effective medium methods

The effective properties of rocks can be estimated by one of the effective medium methods, such as the Mori-Tanaka^1–3^ or the self-consistent method^4^. For all the effective media methods, some assumptions must invariably be made about the shape of the pores, but the size of the pores does not affect the results obtained from the calculations. Typically, the pores are assumed to have an ellipsoidal shape.

A multi-phasic system consisting of $N$ randomly oriented thin spheroidal pores is considered first. Let $a_{1} = a_{2} ,\;a_{3}$ be the semi-axes of the spheroidal cavity (pore) with $a_{3} \ll a_{1}$. We define the aspect ratio of the spheroidal pore as $\rho = a_{3} /a_{1} \ll 1$ (i.e. resembling an oblate spheroidal cavity). The total volume fraction of the pores (i.e. the ratio of the volume of the voids to the total volume) is denoted by $n$. The volume fraction of the pores filled with fluid is $nS$, where $S$ is the degree of saturation. The volume fraction of the pores filled with air is $n(1 - S)$. The bulk moduli of the fluid and the solid phase are denoted by *K*_*f*_ and $K_{s}$, respectively, whereas the bulk modulus of air is $K_{a} \ll K_{f}$. The overall bulk modulus $K_{u}$ of such a three-phase medium in the undrained state can be expressed as

1 $$K_{u} = K_{s} + nSA_{f} (K_{f} - K_{s} ) + n(1 - S)A_{a} (K_{a} - K_{s} )$$

where $A_{f}$ and $A_{a}$ are the total strain localization factors for the fluid phase and the air phase, respectively. In the Mori–Tanaka method the strain localization factors are approximated by the following expressions (Benveniste^21^),

2 $$A_{f} = T_{f} [1 - n + nST_{f} + n(1 - S)T_{a} ]^{ - 1} \;\;A_{a} = T_{a} [1 - n + nST_{f} + n(1 - S)T_{a} ]^{ - 1}$$

where $T_{f}$, $T_{a}$ are, respectively, the partial strain localization factors for the fluid phase and the air. For flat oblate spheroidal pores, the partial strain localization factors take the form (Berryman^22^, Benveniste^21^) (The term strain localization should not be misconstrued for the term that indicates the development of material instabilities.)

3 $$T_{f} = \frac{{K_{s} + 4G_{f} /3}}{{K_{f} + (4/3)G_{f} + \pi \rho \beta_{1} }}\;\;T_{a} = \frac{{K_{s} + 4G_{a} /3}}{{K_{a} + (4/3)G_{a} + \pi \rho \beta_{1} }}$$

and

$$\beta_{1} = G_{s} \frac{{3K_{s} + G_{s} }}{{3K_{s} + 4G_{s} }}$$

The shear modulus of the fluid and the air $G_{f}$, $G_{a}$ in (3) can be set to zero. Note that the results (3) are accurate only if the aspect ratio of the spheroid becomes very small, i.e., $\rho \to 0$. For a two-phase system, in which the saturation $S$ is equal to 1, it is possible to simplify the result (1) to

4 $$K_{u} = K_{s} + n(K_{f} - K_{s} )\left( {\frac{{K_{s} }}{{K_{f} + \pi \rho \beta_{1} }}} \right)\left( {1 - n + \frac{{nK_{s} }}{{K_{f} + \pi \rho \beta_{1} }}} \right)^{ - 1} .$$

The overall bulk modulus of the drained medium is obtained by setting the bulk modulus of the fluid phase $K_{f}$ in (4) equal to zero, which gives

5 $$K_{D} = K_{s} - n\frac{{K_{s}^{2} }}{{\pi \rho \beta_{1} }}\left( {1 - n + \frac{{nK_{s} }}{{\pi \rho \beta_{1} }}} \right)^{ - 1} .$$

By defining the crack density parameter as

6 $$\eta = Na_{1}^{3} /V = \frac{3n}{{4\pi }}\frac{{a_{1} }}{{a_{3} }} = \frac{3n}{{4\pi \rho }}$$

where $N$ is the number of pores in volume $V$, and using the identity

7 $$\frac{{(1 - \nu_{s}^{2} )}}{{(1 - 2\nu_{s} )}} = \frac{{3K_{s} (3K_{s} + 4G_{s} )}}{{4G_{s} (3K_{s} + G_{s} )}}$$

where $\nu_{s}$ is the Poisson ratio of the solid phase, we can use (5) to obtain the following expression for the overall bulk modulus of the drained porous medium $K_{D}$:

8 $$\frac{{K_{s} }}{{K_{D} }} = \left[ {1 + \frac{{16(1 - \nu_{s}^{2} )}}{{9(1 - 2\nu_{s} )}}{\kern 1pt} \,\frac{\eta }{{1 - (4\pi {\kern 1pt} /3)\eta \rho }}} \right].$$

The shear modulus of the drained porous medium $G_{D}$ can be found by using appropriate strain localization factors for shear strain. The result is (Berryman^22^; Benveniste^21^)

9 $$\frac{{G_{s} }}{{G_{D} }} = \left[ {1 + \frac{{32(1 - \nu_{s} )(5 - \nu {}_{s})}}{{45(2 - \nu_{s} )}}\frac{\eta }{{1 - (4\pi {\kern 1pt} /3)\eta \rho }} + \frac{1}{5}\frac{\eta \rho (4\pi /3)}{{\{ 1 - \eta \rho (4\pi /3)\} }}} \right].$$

By taking the limit $\rho \to 0$, the result obtained by Benveniste^21^ for the composite system with very flat penny-shaped cracks can be recovered.

For pores having a *spherical* shape, the partial strain localization factors are given by (Bary^1^)

10 $$T_{f} = \frac{{4G_{s} + 3K_{s} }}{{4G_{s} + 3K_{f} }}\;\;T_{a} = \frac{{4G_{s} + 3K_{s} }}{{4G_{s} + 3K_{a} }}.$$

The formula (1) can now be used to find the overall bulk modulus of the three-phase porous medium having spherical pores. For a two-phase system, in which saturation $S = 1$, the Mori–Tanaka estimate of the overall bulk modulus can be found from (1) as

11 $$K_{u} = K_{s} + n(K_{f} - K_{s} )\left( {\frac{{4G_{s} + 3K_{s} }}{{4G_{s} + 3K_{f} }}} \right)\left[ {1 - n + n\frac{{4G_{s} + 3K_{s} }}{{4G_{s} + 3K_{f} }}} \right]^{ - 1}$$

Consequently, if one sets $K_{f} = 0$ in (11) the overall bulk modulus of the fully drained medium or the skeletal value $K_{D}$ can be obtained.

The self-consistent estimate of the overall properties of the drained porous medium with spherical pores can be obtained from formulae developed by Hill^23^: i.e.

12 $$\frac{1}{{K_{D} + (4/3)G_{D} }} = \frac{n}{{(4/3)G_{D} }} + \frac{1 - n}{{K_{s} + (4/3)G_{D} }}$$

13 $$\frac{1}{{5G_{D} }}\left( {3 - \frac{{K_{D} }}{{K_{D} + (4/3)G_{D} }}} \right) = \frac{1 - n}{{G_{D} }} + \frac{n}{{G_{D} - G_{s} }}$$

Finally, we now assume that the pores of the geomaterial have a needle-like shape, i.e., $a_{3} \gg a_{1}$, $a_{1} = a{}_{2}$. We can use the self-consistent method to obtain estimates for the effective properties of a geomaterial with long fibrous pores randomly distributed within the solid phase. We assume that fully drained conditions prevail. Using the result of Walpole^24^ we can write

14 $$\begin{gathered} K_{D} = (1 - n)K_{s} \left[ {1 + \frac{{nK_{s} }}{{G_{D} }}} \right]^{ - 1} \hfill \\ G_{D} = (1 - n)G_{s} \left[ {1 + \frac{7}{15}\frac{{nG_{s} }}{{G_{D} }} + \frac{2}{5}\frac{{nG_{s} }}{\gamma }} \right]^{ - 1} \hfill \\ \gamma = G_{D} \left( {\frac{{3K_{D} + G_{D} }}{{3K_{D} + 7G_{D} }}} \right) \hfill \\ \end{gathered}$$

## Applications—the Biot coefficient

**Figure 1:**
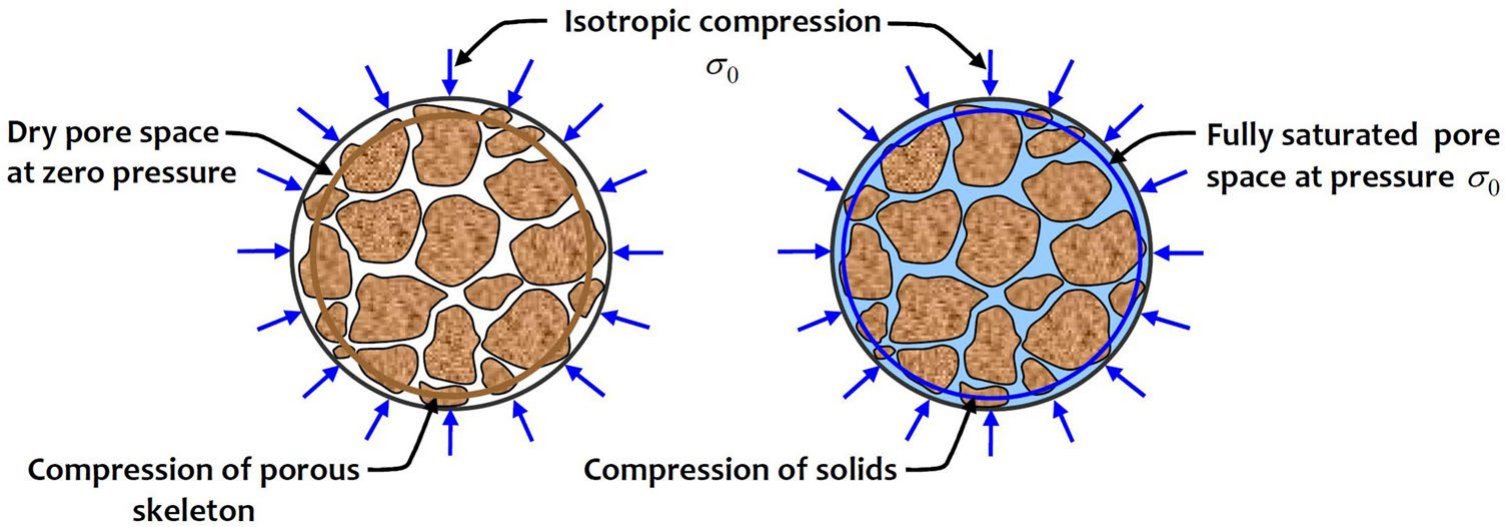
The experimental configurations for estimating the Biot coefficient.

The classical theory of poroelasticity developed by Biot^25^ is an important development with applications in the fields of geoscience and geomechanics, ranging from energy resources extraction, deep geologic disposal of hazardous materials and transport of environmental pollutants, etc. The range of applications of the basic approach developed by Biot^25^ to fields that encompass multi-physics influences arising from temperature change include deep geologic disposal of heat-emitting nuclear waste, geologic sequestration of greenhouse gases, geothermal energy extraction, enhanced oil recovery through steam stimulation, flash heating during earthquake fault rupture and the impact of glaciation on geologic repositories. Recent advances associated with these topics and their extension to multi-porosity materials are documented by Selvadurai and Nguyen^26^, Khalili and Valliappan^27^, Khalili and Selvadurai^28^, Svanadze^29^, Svanadze and De Cicco^30^, Selvadurai et al.^31^ and Selvadurai and Suvorov^32^. The Biot coefficient is an important contribution resulting from the theory of poroelasticity and addresses the issue of partitioning the externally applied stresses between the porous skeleton and the pore fluid. The estimation of the Biot coefficient presents an experimental challenge when the porous medium has low permeability, which makes the process of saturating the pore space in an experimental context both time consuming and unreliable. The complete saturation of the pore space is a necessary pre-requisite for determining the compressibility of the solid materials composing the porous skeleton (Fig. 1).

Alternative approaches that rely on theories of elasticity for multiphasic composites have recently been applied to overcome this impediment (Selvadurai^33^; Selvadurai et al.^34^). The results derived previously can now be used to obtain an estimate of the Biot coefficient if one of the elastic moduli, either the bulk modulus of the skeleton $K_{D}$ or the bulk modulus of the solid phase $K_{S}$ is known but the other is not. The Biot coefficient is defined by

15 $$\alpha = 1 - \frac{{K_{D} }}{{K_{S} }}$$

where $K_{D}$ and $K_{S}$ are, respectively, the bulk moduli values for the porous skeleton and the solid material. If the constitutive behaviour of the porous skeleton deviates from elastic behaviour, the approach used to define the Biot coefficient will change (Suvorov and Selvadurai^35^).

**Figure 2:**
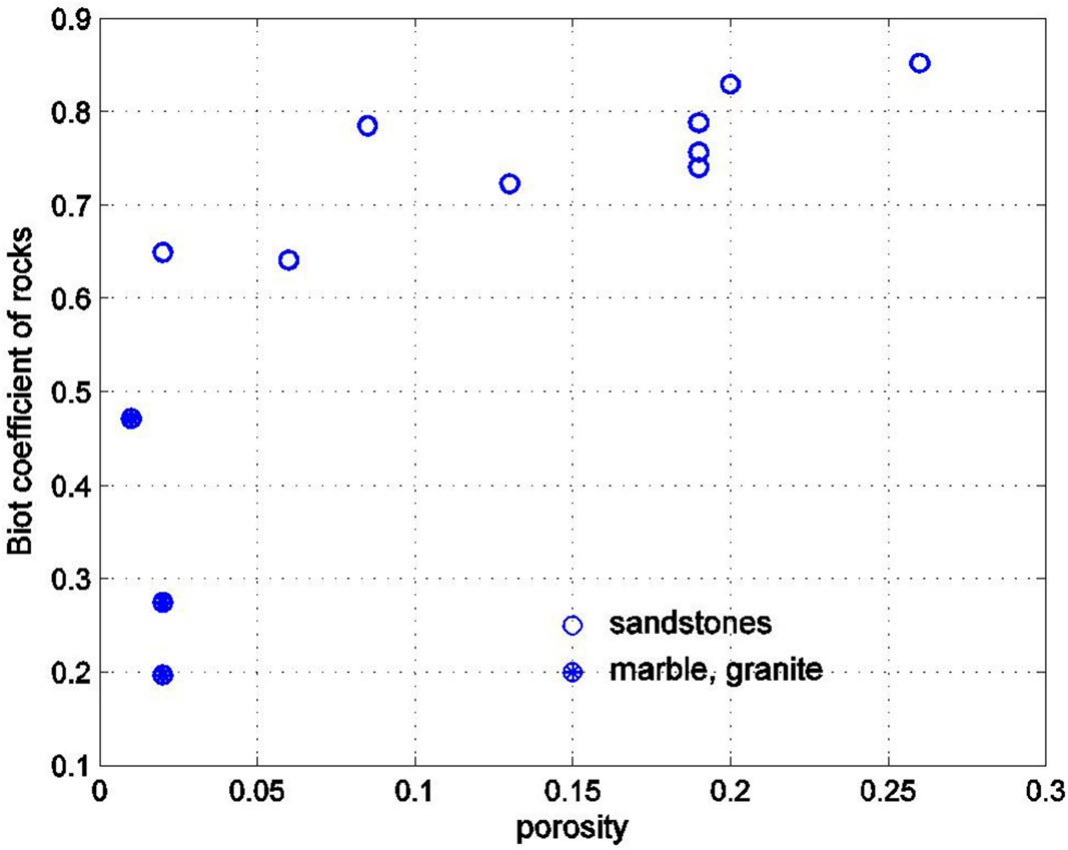
Experimentally measured Biot coefficient versus porosity for sandstones, marble and granite.

Figure 2 shows how the Biot coefficient depends on the porosity of the rock. This data is merely a collection of experimental results presented by Hart and Wang^36^ and Zimmerman^37^. The Biot coefficient for sandstones is shown with empty circles, the Biot coefficient of marble and granite is shown with filled circles. It can be seen that the Biot coefficient for sandstones ranges from 0.6 to 0.9, and, on average, is an increasing function of porosity. [This stands to reason since, as the porosity increases, $K_{D} \ll K_{S}$ and $\alpha \to 1$.] For marble and granite the Biot coefficient is lower than 0.5.

This data is now analyzed from a micromechanics point of view. Using the effective medium methods, the effective elastic properties can be estimated from the given phase properties or vice versa, the phase properties can be estimated given the effective (overall) properties.

Consider the situation where the porosity $n$, the bulk modulus and the shear modulus of the drained porous material or the skeleton $K_{D}$ and $G_{D}$, respectively, are known from experimental data. The properties of the solid phase, in particular the bulk modulus $K_{S}$, and the properties of the rock in an undrained state $K_{u}$ have then to be determined. We now consider the case of thin spheroidal pores.

For linear elastic materials we make use of the relationships (Davis and Selvadurai ^38^)

16 $$\nu_{S} = \frac{{3K_{s} - 2G_{s} }}{{2(3K_{s} + G_{s} )}};\quad K_{S} = \frac{{2G_{s} }}{3}\frac{{1 + \nu_{s} }}{{1 - 2\nu_{s} }}$$

The Eqs. (16), (8) and (9) have then to be solved for the unknown elastic properties of the solid phase $K_{S}$, $G_{S}$, $\nu_{S}$.

For simplicity, we set the aspect ratio $\rho = 0$ in (8), (9), although more accurate results can be obtained when $\rho \ne 0$. First, we can express $K_{D}$ using (8) and the connection (16), which gives

17 $$K_{D} = \frac{{2G_{S} }}{3}\frac{{1 + \nu_{s} }}{{1 - 2\nu_{s} + (16/9)\eta (1 - \nu_{s}^{2} )}}$$

Therefore, from (17)

18 $$G_{S} = \frac{{3K_{D} }}{2}\frac{{1 - 2\nu_{s} + 16\eta (1 - \nu_{s}^{2} )/9}}{{1 + \nu_{s} }}$$

The result (18) can now be used in (9) to give

19 $$\frac{{G_{S} }}{{G_{D} }} = \frac{{3K_{D} }}{{2G_{D} }}\frac{{1 - 2\nu_{s} + 16\eta (1 - \nu_{s}^{2} )/9}}{{1 + \nu_{s} }} = \left( {1 + \frac{{32(1 - \nu_{s} )(5 - \nu {}_{s})}}{{45(2 - \nu_{s} )}}\eta } \right)$$

The non-linear Eq. (19) can be solved either analytically or numerically to determine Poisson’s ratio for the solid phase $\nu_{S}$. Note that the Poisson’s ratio must satisfy the energetic constraint $\nu_{D} \le \nu_{S} \le 0.5$, i.e., it is expected to be larger than the Poisson’s ratio of the drained medium $\nu_{D}$ but less than 0.5. Using the known Poisson’s ratio of the solid phase $\nu_{S}$, the bulk and shear moduli and $K_{S}$, and $G_{S}$ respectively, of the solid phase can be found from (8), (9). Note that the relation (16) will be satisfied. The Biot coefficient $\alpha$ can now be found from (15).

**Figure 3:**
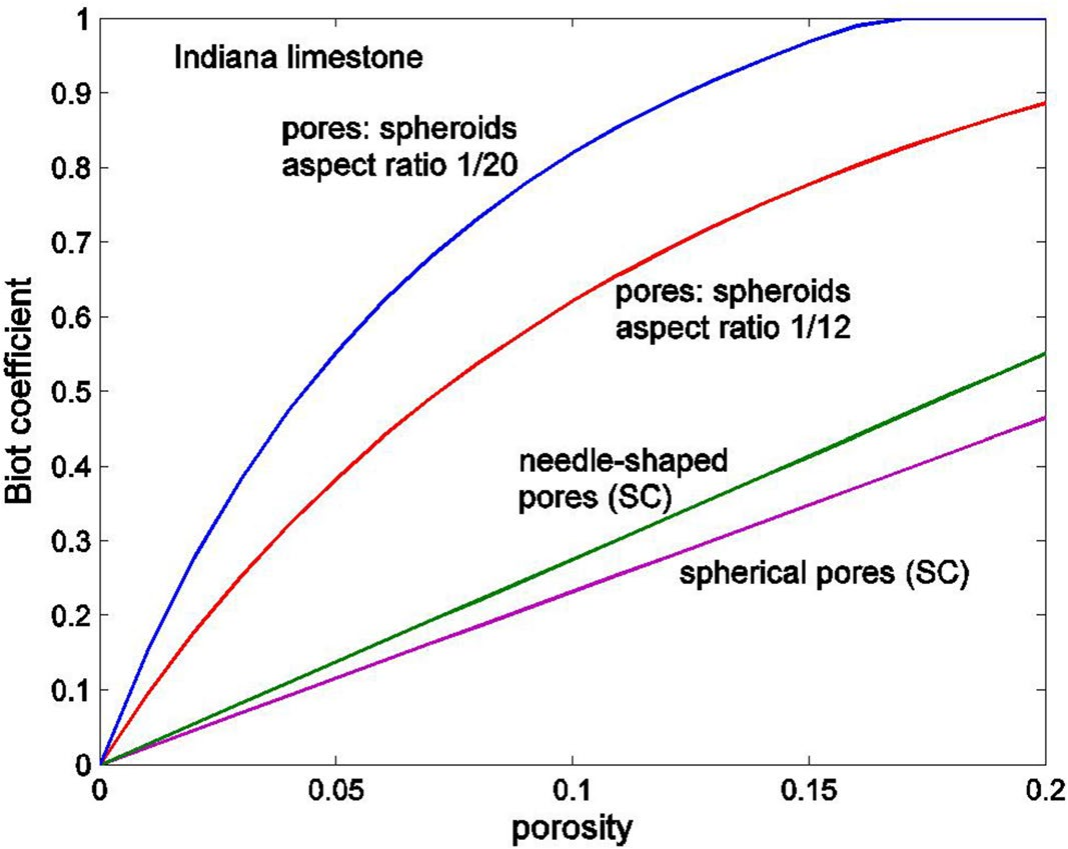
Approximate estimates of the Biot coefficient as a function of the porosity. Estimates were obtained using the Mori–Tanaka method for a rock with flat ellipsoidal pores and the self-consistent method (SC) was used for a rock with spherical and needle-shaped pores. The elastic properties of the rock are those of the Indiana limestone.

Figure 3 shows both the Mori–Tanaka and self-consistent estimates of the Biot coefficient for geomaterials with the elastic properties of Indiana limestone, $K_{D} =$ 21.2 GPa, $G_{D} = 12.11$ GPa, $\nu_{D} = 0.26$ (Hart and Wang^36^). The Mori–Tanaka method is used when the pores are oblate spheroids (8), (9), and the self-consistent (SC) method (12)–(14) is used when the pores are either spheres or needle-shaped. It can be seen that for rocks with spherical or needled-shaped pores, the Biot coefficient is much lower than for rocks with pores that have a thin spheroid shape. Also, with the decrease in aspect ratio of the oblate spheroids, the Biot parameter approaches its upper limit equal to 1. For Indiana limestone with a porosity $n = 0.13$, the experimentally measured value of the Biot coefficient is equal to 0.708 (Hart and Wang^36^). A good match between the experimentally measured value and the analytical result can be obtained if the aspect ratio of the spheroids is set equal to 1/12.

**Figure 4:**
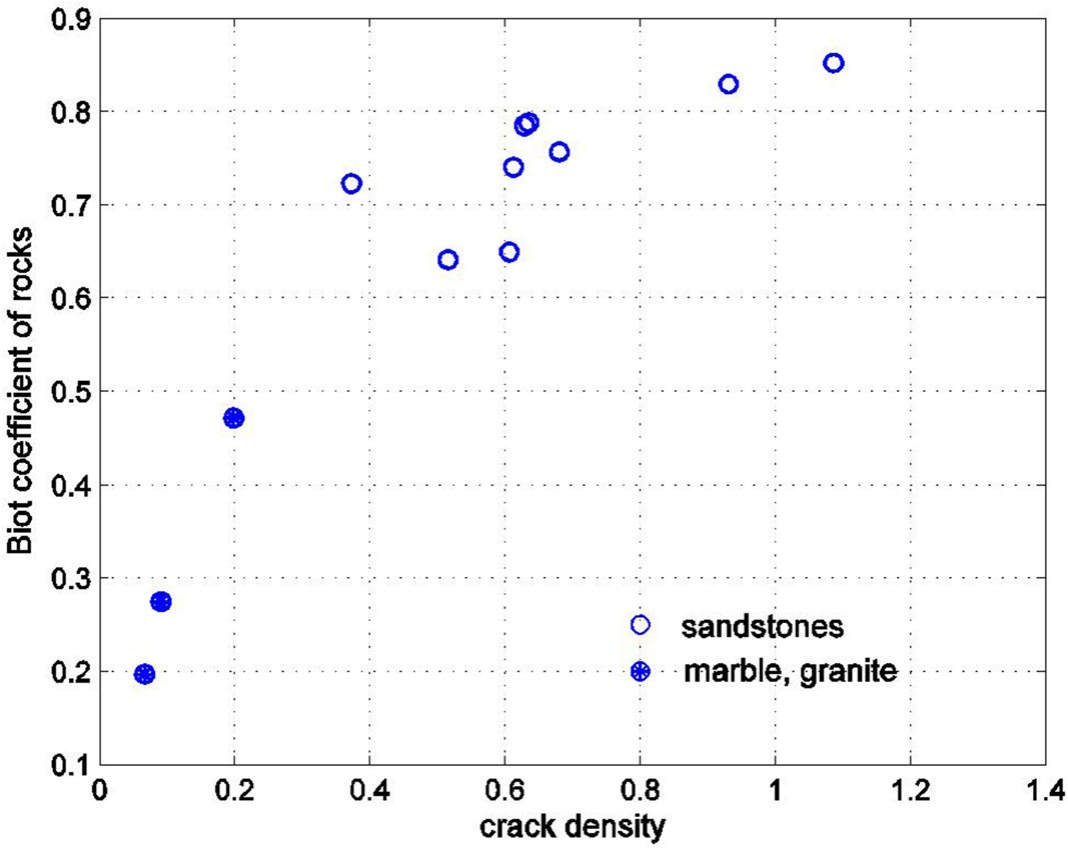
Dependence of the Biot coefficient on the crack density parameter for rocks shown in Fig. 2. The crack density parameter was found by matching the experimentally measured Biot coefficient with its Mori–Tanaka estimate.

It is now possible to match the results of Figs. 2 and 3 if the pores are assumed to be oblate spheroids with a small aspect ratio. By varying the aspect ratio, or the crack density parameter, the best agreement with the experimentally measured values of the Biot coefficient can be found. The results of comparisons appear in subsequent Figures. Figure 4 shows the dependence of the Biot coefficient of the rocks on the crack density parameter $\eta$

20 $$\eta = Na_{1}^{3} /V = \frac{3n}{{4\pi \rho }}.$$

**Figure 5:**
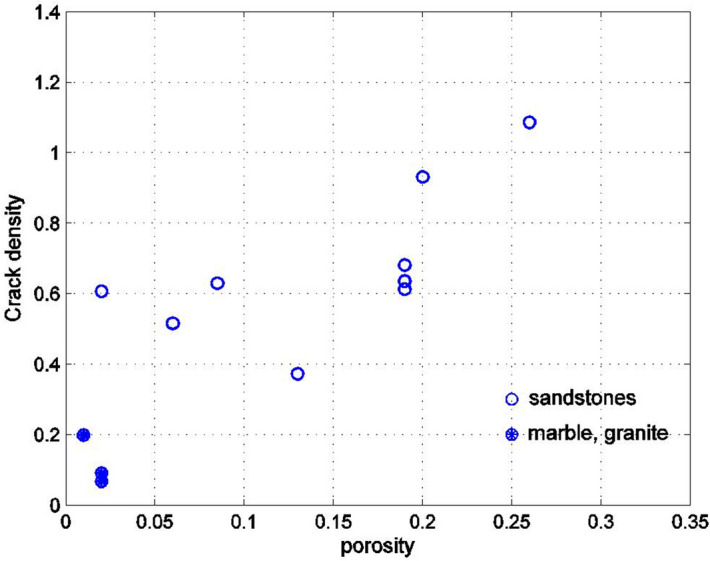
Dependency of the crack density parameter, shown in Fig. 4 on the porosity for rocks in Fig. 2.

**Figure 6:**
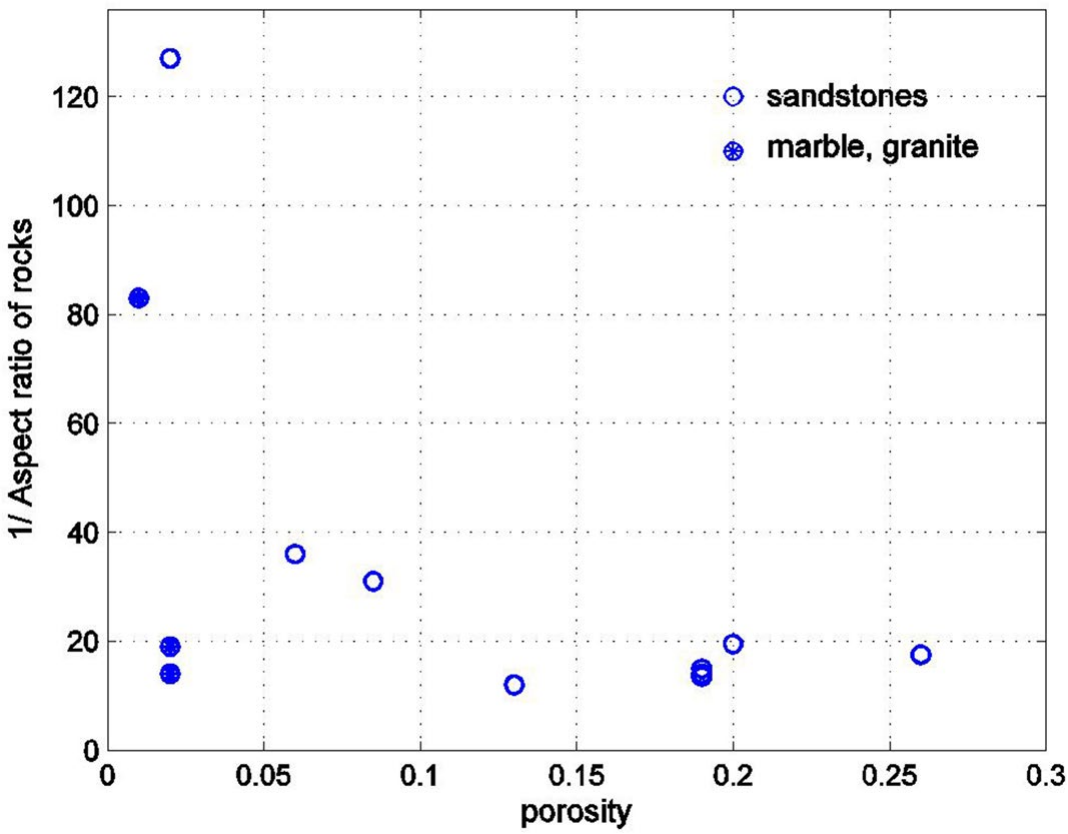
Variation of the inverse of the aspect ratio of the spheroidal pores with the porosity of the rocks in Fig. 1. The aspect ratio was estimated by matching the experimentally measured Biot coefficient with its Mori–Tanaka estimate.

As can be seen, the Biot coefficient increases with the crack density (i.e., the presence of the cracks reduces the skeletal stiffness), and for sandstones the crack density is larger than for marble and granite. The graph does not have a great deal of scatter, which shows that the Poisson’s ratio of the material of the solid phase in the rock is approximately constant (for a constant Poisson’s ratio of the solid phase and very small aspect ratios of the pores $\rho$, the Biot coefficient is a smooth function of the crack density $\eta$, which follows from (8)). It would be difficult to use this graph in practice since the crack density parameter is not usually known from experimental data. In fact, both the porosity $n$ and the aspect ratio of the spheroid $\rho = a_{3} /a_{1}$ are needed to determine the crack density $\eta$. The porosity $n$ is usually known from experimental data; thus, finding the dependency of the crack density $\eta$ on the porosity is a useful result. The dependence of the aspect ratio $a_{3} /a_{1} \ll 1$ of the oblate spheroids on the porosity should also be considered. Figure 5 shows the variation of the crack density on porosity and Fig. 6 shows the variation of the inverse of the crack density on porosity. For sandstones, the crack density appears to be roughly constant, equal to about 0.5 for porosities smaller than 0.15 (Fig. 5). For the porosities greater than 0.15, the crack density begins to increase, reaching values above 1 for large porosities. For marble and granite, the crack density is considerably smaller than for sandstones.

From Fig. 6 we can see that the aspect ratio of the oblate spheroidal pores is small, remaining roughly equal to between 1/15 and 1/20 for porosities greater than 0.1. However, in sandstones with porosities lower than 0.1, the aspect ratio of the pores decreases (inverse of the aspect ratio increases). For marble and granite, the aspect ratio is larger than for sandstones. By matching the measurements of the effective conductivity of rocks with the theoretical estimates, Gruescu et al.^3^ reported that the aspect ratio of the spheroidal pores should be on the order of 0.05.

The bulk modulus of the undrained fully-saturated rock can be found from the Biot–Gassmann equation (Gassmann^39^; Saxena^40^)

21 $$K_{u} = K_{D} + \frac{{\alpha^{2} }}{{\frac{n}{{K_{f} }} + \frac{\alpha - n}{{K_{s} }}}}.$$

Note that the Biot–Gassmann equation is only valid for an isotropic porous medium with arbitrary pore shapes. For very thin spheroidal pores, the bulk modulus of the undrained medium can also be found using the Mori–Tanaka estimate (8) derived previously. The results will be equal if the aspect ratio $\rho$ of the pores is assumed to be small.

For a three-phase partially saturated medium, the Mori–Tanaka estimates can also be used to determine the equivalent bulk modulus of the fluid–gas mixture $K_{fa}$ as a function of the saturation $S$ and the fluid bulk modulus $K_{f}$. In particular, equating the bulk modulus of the three-phase porous medium (1) to the bulk modulus of the two-phase medium (4) with the fluid having equivalent properties $K_{fa}$, gives

22 $$\begin{aligned} & nSA_{f} (K_{f} - K_{s} ) + n(1 - S)A_{a} ( - K_{s} ) \\ & \quad = n(K_{fa} - K_{s} )\left( {\frac{{K_{s} }}{{K_{fa} + \pi \rho \beta_{1} }}} \right)\left[ {1 - n + n\frac{{K_{s} }}{{K_{fa} + \pi \rho \beta_{1} }}} \right]^{ - 1} \\ \end{aligned}$$

where the bulk modulus of the gaseous phase was set to zero. The porosity $n$ can be expressed in terms of the aspect ratio and crack density using (6), $n = 4\pi \eta \rho /3$. Equation (22) can now be solved for $K_{fa}$. Alternatively, one could use the Biot–Gassmann result (21) for the bulk modulus of the two-phase medium with an equivalent fluid phase. This gives the following equation:

23 $$nSA_{f} (K_{f} - K_{s} ) + n(1 - S)A_{a} ( - K_{s} ) = K_{D} + \frac{{\alpha^{2} }}{{\frac{n}{{K_{fa} }} + \frac{\alpha - n}{{K_{s} }}}}$$

**Figure 7:**
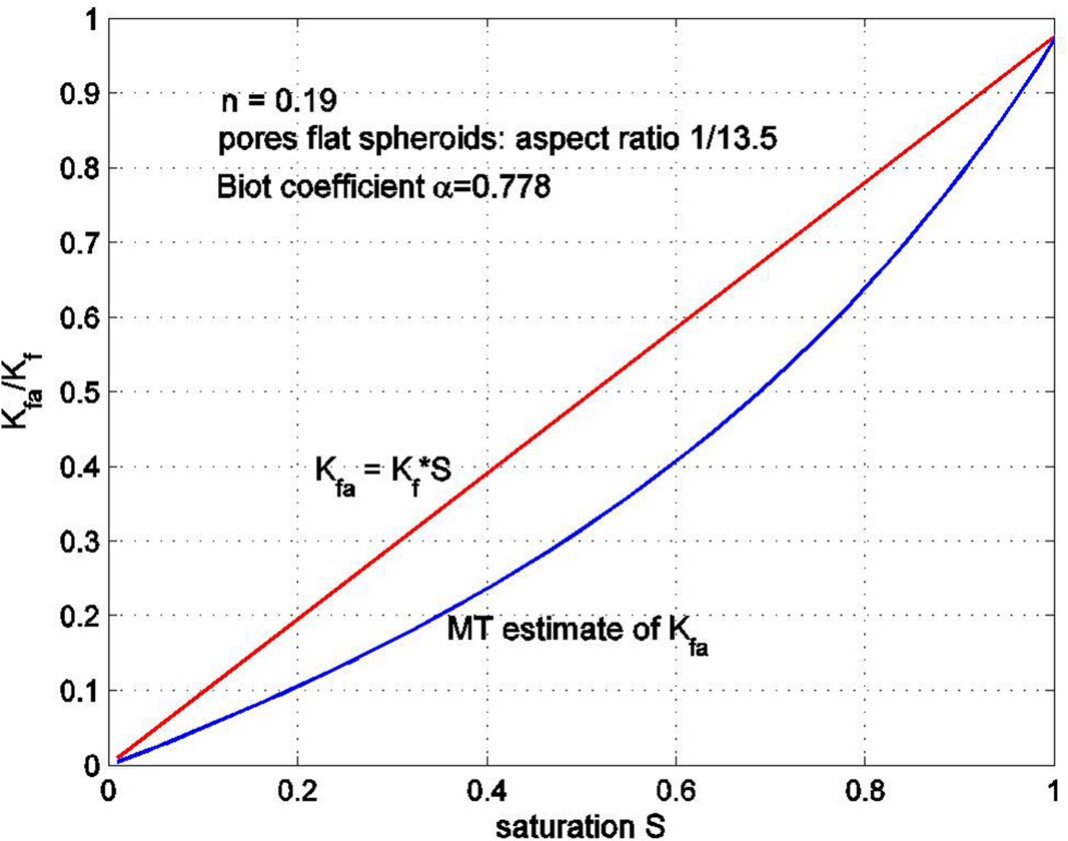
Normalized bulk modulus of the fluid–air mixture as a function of saturation. The pores are flat spheroids with an aspect ratio 1/13.5. The Mori–Tanaka (MT) estimate was obtained from the estimate of the overall bulk modulus for a three-phase porous medium having randomly oriented pores filled with fluid and gas. The bulk modulus of the gas phase is very small compared to that of the fluid.

**Figure 8:**
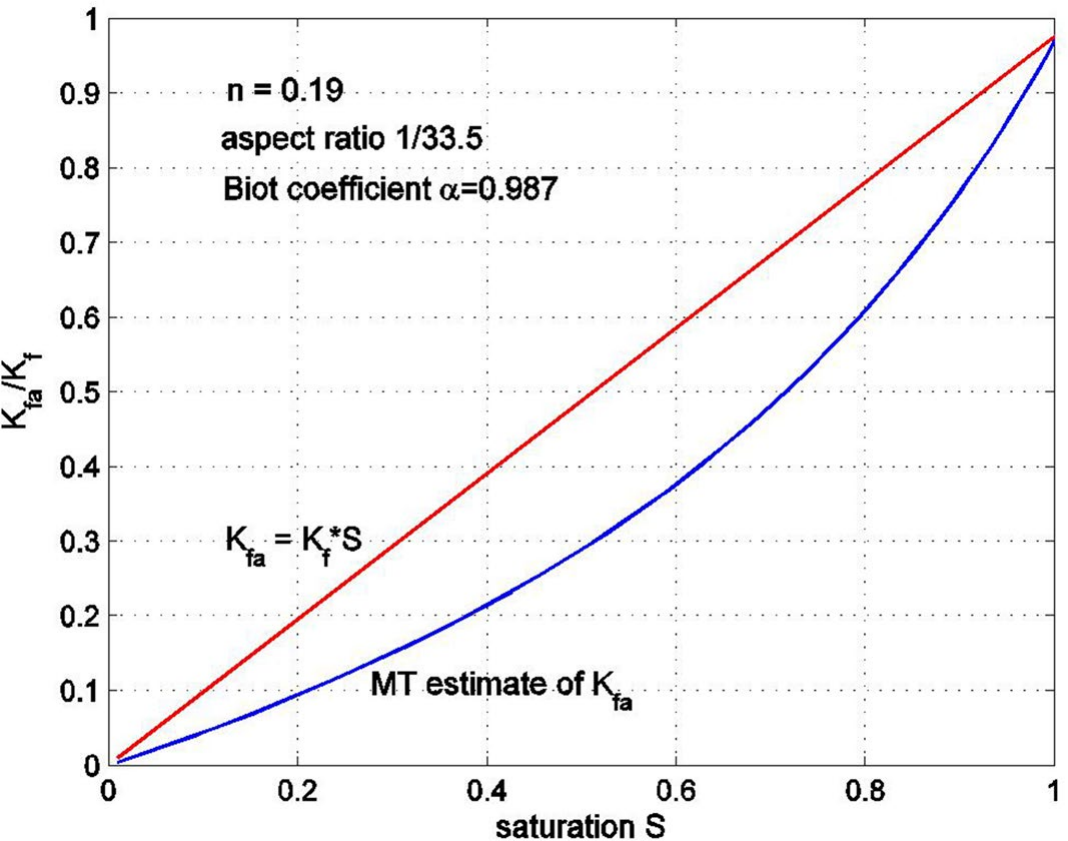
Normalized bulk modulus of the fluid–air mixture as a function of saturation. The pores are flat spheroids with an aspect ratio 1/33.5.

**Figure 9:**
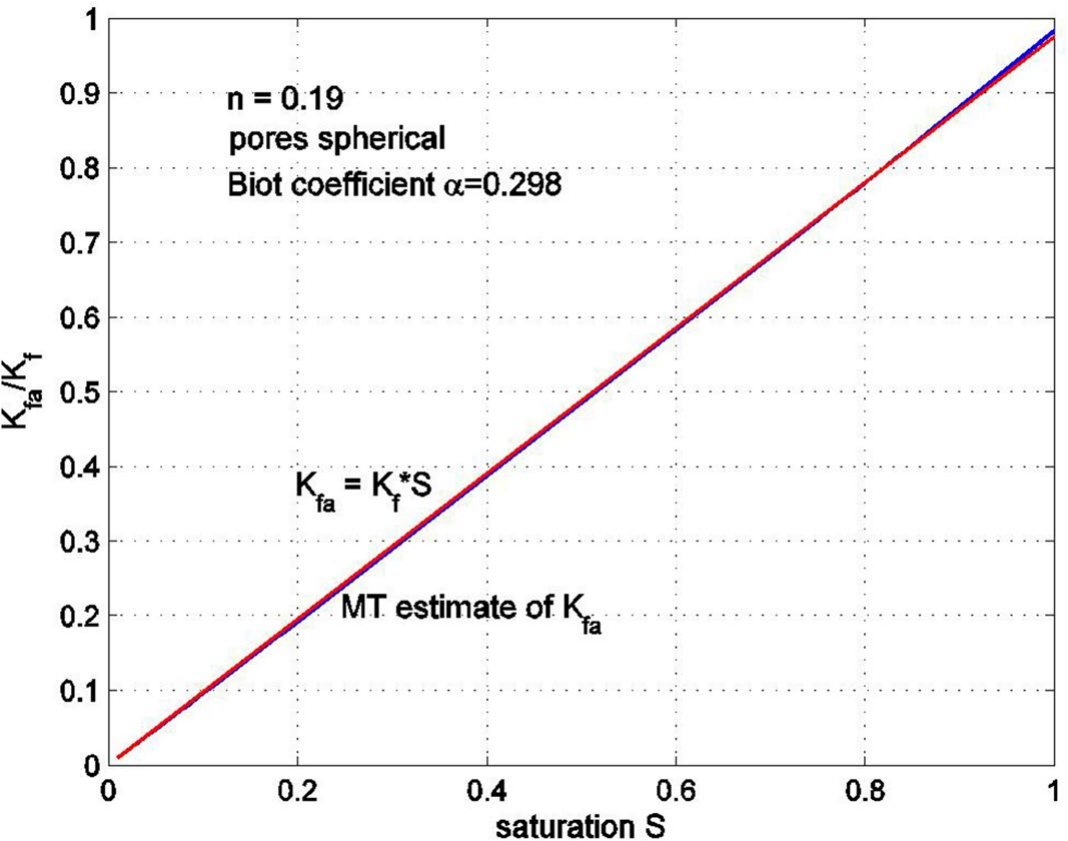
Normalized bulk modulus of the fluid–air mixture as a function of saturation. The pores are spheres.

Equation (23) can be solved for $K_{fa}$. Note that the solutions provided by (22) and (23) will be equal only if the aspect ratio $\rho$ of the spheroidal pore is assumed to be small. Similar equations can be obtained for a porous medium with spherical pores. Figures 7 and 8 show the equivalent bulk modulus of the fluid–gas mixture $K_{fa}$ as a function of the saturation $S$ for the porous medium with spheroidal pores having aspect ratios of 1/13.5 and 1/33.5, respectively. The rock porosity is 0.19, and the properties of the rock constituents are: $K_{f} = 2.2$ GPa, $K_{S} = 29.73$ GPa, $G_{S} = 11.47$ GPa, with the overall bulk modulus of the drained rock $K_{D} = 6.6$ GPa. The results are obtained using the formula (22). The equivalent bulk modulus $K_{fa}$ obtained from formula (22) is compared with the simplified expression $K_{fa} = SK_{f}$. It can be seen that the difference between the two solutions is not very significant, especially for large or small levels of saturation. Note that as the aspect ratio becomes smaller, the equivalent bulk modulus $K_{fa}$ tends to zero and, in the limit $\rho = 0$, when all pores are very wide and thin, $K_{fa} \to 0$. Figure 9 shows the saturation dependency of the equivalent bulk modulus of the fluid–gas mixture $K_{fa}$ for a porous medium with spherical pores, estimated using the Mori–Tanaka method. This estimate is very close to $K_{fa} = SK_{f}$.

## Conclusions

The conventional approaches for the estimation of overall elastic properties of porous media largely focus on a scalar measure of porosity. The results presented in this study clearly demonstrate that the overall elastic properties of a porous medium are also dependent on the shape of the pores. This finding has important implications for examining the stress partitioning between the saturating fluid and the porous skeleton as addressed in Biot’s^25^ theory. The Biot coefficient for most rocks lies in the range 0.6–0.8 and the porosity of rocks is usually smaller than 0.2. It is possible to match the experimentally measured Biot coefficient with the analytical estimates if the pores are assumed to be thin oblate spheroids. It was found that the aspect ratio of the spheroids should decrease as the porosity gets smaller, but for porosities larger than 0.1, the aspect ratio is found to be equal to about 1/20 for most sandstones.

For saturated porous media in particular, the equivalent bulk modulus of the fluid–gas mixture was also found to be dependent on the shape of the pores. In particular, if the bulk modulus of the air is neglected, for spherical pores the equivalent bulk modulus is very close to the estimate $SK_{f}$, For thin oblate spheroids, the equivalent bulk modulus decreases as the aspect ratio becomes smaller. Even for an aspect ratio of 1/33, the Mori–Tanaka estimate of the equivalent bulk modulus is not significantly different from $SK_{f}$.
